# Health State Utility Values in Children and Adolescents with Disabilities: A Systematic Review

**DOI:** 10.1016/j.jpedcp.2025.200139

**Published:** 2025-01-15

**Authors:** Lucy Kanya, Nana Anokye, Ahmad Hecham Alani, Nandini Jayakumar, Jennifer M. Ryan

**Affiliations:** 1 Department of Health Policy, London School of Economics and Political Science, London, United Kingdom; 2 Institute of Environment, Health and Societies, Brunel University, London, United Kingdom; 3 Department of Sociology, University of Cambridge, Cambridge, United Kingdom; 4 School of Physiotherapy, RCSI University of Medicine and Health Sciences, Dublin, Ireland

**Keywords:** health utilities, preference-based measures, utility measurement methods, disability, pediatric disabilities, preference elicitation, psychometric evaluation

## Abstract

**Objectives:**

To (1) provide a comprehensive summary of the methods used to obtain health state utility values (HSUVs) from children and adolescents with disabilities (CAD), (2) describe the administration and psychometric properties of these methods in children and adolescents with disabilities, and (3) report summary statistics for HSUVs obtained from each method.

**Study design:**

English-language studies from MEDLINE (via PubMed), PsychInfo, Scopus, CINAHL Plus, EconLit, and Embase were searched from inception to November 2024. Two reviewers independently screened titles, abstracts, and full texts. Studies were included if they used direct or indirect methods to measure HSUVs, reported utilities and/or psychometric properties of these measures, and involved CAD aged 0-19 years. Two reviewers independently extracted study details including sample descriptors, instruments used, and summary statistics. Studies quality was assessed using a novel tool derived from 3 validated checklists.

**Results:**

Of the 3541 screened articles, 31 met inclusion criteria. Only 2 studies used direct methods, such as time trade-off, visual analog scale, and standard gamble, whereas 29 employed generic measures (eg, EuroQol 5 Dimensions, Health Utilities Index 3) with diverse preference elicitation methods. Excessive dependence on proxy respondents was noted, and psychometric properties of generic measures were mixed.

**Conclusions:**

Inconsistent HSUVs reporting and limited data availability are common. Reported HSUV summary statistics may be inaccurate if methodologies are unsuitable for the population. This review emphasizes the need for validated instruments to assess HSUVs in CAD.

Disability is defined as a challenge in functioning across body, personal, or societal levels, stemming from the interaction between an individual's health condition and contextual factors such as negative attitudes, inaccessible buildings, and lack of social support.^1^^,^^2^ Approximately 5% of children worldwide experience moderate or severe disability,^2^ necessitating research on effective interventions to enhance activity and participation outcomes.^3^ Decisions on adopting health care interventions typically are informed by evaluations of cost-effectiveness, where net costs are assessed in the context of improvement in health outcomes. Cost-utility analysis (CUA) is a common approach for evidence-based decision-making in health care interventions, comparing costs with quality-adjusted life years. Quality-adjusted life years measure the quantity of life years and the quality of life using health state utility values (HSUVs), ranked from 0 (indicating a state equivalent to being dead) to 1 (representing full health). HSUVs can be obtained through 2 broad categories of methods. Direct methods, like time trade-off (TTO) and standard gamble (SG), engage individuals in assessing and assigning scores to health states.^4^ Indirect methods use generic measures of health-related quality of life (eg, EuroQol 5 dimensions questionnaire [EQ-5D]) and derive utility values using scoring algorithms on the basis of preferences from the general population.^4^ Generic measures typically are recommended for use in economic evaluations, as they allow for comparison across different health conditions.^5^ Data collection for CUA should be robust, transparent, and systematic to enhance evidence reliability.^5^^,^^6^ However, reviews identified potential validity issues with generic HSUVs measures in adults with physical disabilities.^7^^,^^8^

Limited information is available on HSUVs among children and adolescents with disabilities (CAD), including the psychometric properties of measures in this population. Understanding these methods is crucial for interpreting CUA findings and informing research and practice. The objectives of this review are to describe methods used to obtain HSUVs in CAD, including how they are administered, describe psychometric properties of these methods in CAD, and report summary statistics for HSUVs among CAD obtained from each method.

## Methods

The study design was informed by published recommendations for reviewing HSUVs.^9^, ^10^, ^11^ The protocol for this review was registered with the International Prospective Register of Systematic Reviews (CRD42018086574) and published.^12^ Reporting of the review adhered to the PRISMA guidelines.^13^

### Search Strategy

We conducted a comprehensive search of the following databases from inception to September 3, 2023: MEDLINE (via PubMed), PsychInfo, Scopus, CINAHL Plus, EconLit, and Embase. The search was updated to include studies published up to November 8, 2024. Reference lists of key papers also were screened for additional references. The search strategy was developed on the basis of a pilot search of the literature and included various combinations of key words and subject headings related to children and adolescents (eg, infant, newborn, child, and adolescent), health utility terms (eg, EQ-5D, TTO, SG), and disability terms (eg, disabled, impairment). The search strategies were adapted for each database. An example of our search strategy was previously published.^12^

### Eligibility Criteria

We included studies of any design that were reported in English and (1) reported HSUVs among CAD derived from both direct (eg, SG, TTO, visual analog scale [VAS]) and indirect methods (eg, EuroQol EQ-5D and its variants, Child Health Utility 9D [CHU-9D], Assessment of Quality of Life [AQoL], Health Utilities Index [HUI], and Quality of Well-Being [QWB]. among others.); and/or (2) reported the psychometric properties of measures used to obtain HSUVs in CAD; and (3) included CAD aged 0-19 years. Studies that included adolescents and adults with disabilities also were included if data could be extracted for adolescents separately or if the overall mean age of the sample was <18 years. We included studies involving children and adolescents with intellectual impairment, physical impairment, developmental disability, sensory impairments, and multiple impairments. We excluded reviews, commentaries, unpublished theses, and conference abstracts.

### Data Screening and Extraction

Screening and data extraction were completed by 4 reviewers. Titles and abstracts were screened independently by 2 reviewers. The full texts of potentially eligible studies were obtained and independently screened by 2 reviewers. Data extraction was conducted independently by 2 reviewers. Disagreements between the reviewers were resolved through discussion.

We used a standardized form to extract data on study aims and methods including study design, setting, sampling method; sample characteristics including age, sex, race, socioeconomic status, diagnosis, type of disability, disability severity; methods used to obtain HSUVs including instrument, mode of administration, data source, time points, length of time to complete or administer; psychometric properties of the instrument in CAD with disabilities including validity, reliability, responsiveness; and summary statistics for HSUVs. This form was created and piloted by 2 reviewers. The International Society for Quality of Life Research minimum standards for patient-reported outcome measures guided the data-extraction items, including information on reliability, validity, and burden of patient-reported outcome measures.^14^ In addition, select data extraction items from the Checklist for REporting VAluaTion StudiEs were used, such as description of instrument attributes, sampling method, response rate, and reasons for excluding respondents or observations.^15^ Because of the broad objectives of this review, not all data extraction items on the Checklist for REporting VAluaTion StudiEs checklist were applicable to all included studies.

### Quality Assessment

Quality assessment was conducted independently by 2 single reviewers and disagreements resolved through discussion. Because of the absence of an existing suitable checklist, reviewers independently assessed study quality using a novel checklist derived from 3 sources. The first source was the Standards of the Systematic Review of Utilities for Cost-Effectiveness checklist, created by an ISPOR Good Practices for Outcomes Research Task Force and which provides recommendations for synthesizing HSUVs for cost-effectiveness models.^16^ The second source offered guidance on systematic literature review for HSUVs identification/selection.^9^ The third source was a quality appraisal analysis of systematic literature reviews for HSUVs.^17^ The derived checklist encompassed items such as study population, inclusion/exclusion criteria, administration details (eg, responder, assessor training), sample size, response rate, missing data, and discussion of potential bias and generalizability of findings. The checklist was piloted, adjusted as needed prior to its application in the study. Studies were assessed using a 14-item tool, scoring each study on the basis of “yes” (1 point), “somewhat” (0.5 points), or “no/not clear” (0 points) answers. This nuanced scoring considered the extent to which each criterion was met.

### Data Analysis

The characteristics of the included studies, methods used to obtain HSUVs and their administration, and psychometric properties of the methods in CAD were narratively summarized. Summary statistics for HSUVs were reported according to disability type, ie, intellectual impairment, physical impairment, developmental disability, sensory impairment, and multiple impairments. Given the clinical heterogeneity observed in the identified studies, particularly in terms of the type and severity of disability, a narrative synthesis approach was adopted to summarize the findings. Further, on the basis of expert review, the authors determined that providing a single estimate for each condition was not clinically useful nor sufficiently robust, considering the diversity of the identified studies.

## Results

The Figure summarizes the selection process. After we removed duplicates, 3541 titles and abstracts were screened, and 249 full texts obtained for evaluation. Subsequently, 31 studies^18^, ^19^, ^20^, ^21^, ^22^, ^23^, ^24^, ^25^, ^26^, ^27^, ^28^, ^29^, ^30^, ^31^, ^32^, ^33^, ^34^, ^35^, ^36^, ^37^, ^38^, ^39^, ^40^, ^41^, ^42^, ^43^, ^44^, ^45^ were eligible for inclusion in the review. Quality assessment scores ranged from 4 to 12, out of 14 points, with a mean score of 9 (Appendix). Among these studies, 39% (n = 12)^18^^,^^19^^,^^22^^,^^23^^,^^26^^,^^28^^,^^35^^,^^37^^,^^41^^,^^42^^,^^46^^,^^47^ were deemed high quality (scores of 10 or greater), whereas 6 were of lower quality,^21^^,^^25^^,^^38^, ^39^, ^40^^,^^48^ (scoring 7 or lower). Common quality issues included the lack of assessor training in tool administration in 65% of studies (n = 20); missing data without explanation (39%, n = 12); inadequate justification of sample sizes (48%, n = 15), and failure to discuss potential sources of bias, including attempts to minimize bias (45%, n = 14).

**Figure:**
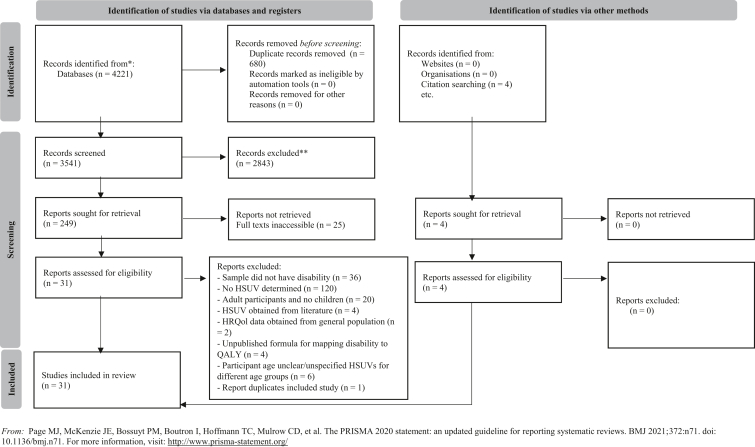
PRISMA 2020 flow diagram.

### Study Characteristics

Table I summarizes the included studies. These were conducted in 18 countries: France, Germany, Italy, Lithuania, the Netherlands, Ireland, Saudi Arabia, Spain, Sweden, United Kingdom, Canada, US, Brazil, China, India, Israel, Thailand, and Australia.^18^, ^19^, ^20^, ^21^, ^22^, ^23^, ^24^, ^25^, ^26^, ^27^, ^28^, ^29^, ^30^, ^31^, ^32^, ^33^, ^34^, ^35^, ^36^, ^37^, ^38^, ^39^, ^40^, ^41^, ^42^, ^43^, ^44^, ^45^ The studies involved 12 663 participants (range: 12-4016). Among them, 24 were cross-sectional studies,^19^, ^20^, ^21^, ^22^, ^23^, ^24^, ^25^, ^26^, ^27^, ^28^, ^29^, ^30^, ^31^, ^32^, ^33^^,^^36^, ^37^, ^38^^,^^40^^,^^44^, ^45^, ^46^, ^47^, ^48^ 6 were randomized controlled trials,^34^^,^^35^^,^^39^^,^^41^, ^42^, ^43^ and 1 was a CUA.^18^ The most commonly assessed conditions were deafness or hearing impairment, (n = 11),^18^^,^^20^, ^21^, ^22^, ^23^, ^24^^,^^28^^,^^30^^,^^36^^,^^38^^,^^40^ cerebral palsy (n = 8),^23^, ^24^, ^25^^,^^29^^,^^39^^,^^40^^,^^44^^,^^45^ and autism spectrum disorders/autism (n = 7).^24^^,^^26^^,^^30^^,^^41^^,^^44^^,^^46^^,^^47^ The age range was reported in 19 studies (61%). Participant ages ranged from 11 months to 18 years in all studies.

**Table I:** Characteristics of the included studies

Authors	Year	Country	Study design	Description of children	Condition(s) covered	Respondent	Measure(s)
Cheng et al^18^	2000	US	CUA	n = 78; mean (SD) age 7.5 (4.5) y; 46% female	Profoundly deaf	Parent	HUI; TTO; VAS
Tilford et al^19^	2005	US	Cross-sectional	n = 98; mean (SD) age 9.3 (4.6) y; range 2-17 years; 61.2% female	Spina bifida	Caregiver	HUI-2
Barton et al^20^	2006	UK	Cross-sectional	n = 2858	Hearing loss	Parent	HUI-3
Sach et al^21^	2007	UK	Cross-sectional	n = 216; mean (SD) age 9.3 (3.6) y; 50.5% female	Hearing loss	Parent	EQ-5D
Rosenbaum et al^45^	2007	Canada	Cross-sectional	n = 203; mean (SD) age 16 (1.8) y; 45.3% female	Cerebral palsy	Child or parent	HUI-3
Smith-Olinde et al^22^	2008	US	Cross-sectional	n = 103; mean (SD) age 7.3 (1.9) y; range 5-10 y; 48.5% female	Hearing loss	Caregiver	HUI-3; QWB
Carroll et al^23^	2009	US	Cross-sectional	n = 4016	Bilateral vision loss; cerebral palsy; hearing loss; “mental retardation”; monocular blindness	Parent	SG; TTO
Petrou et al^24^	2009	UK	Cross-sectional	n = 2236; age range 5-16 y; 50.4% female	Autism spectrum disorders; learning disabilities; severe learning disabilities/global developmental delay; learning and physical disabilities; Down syndrome; cerebral palsy; unspecified motor disorders; head injury; vision disorders and blindness; deafness; deafness with other impairments; speech disorders	Caregiver	HUI-3
Young et al^25^	2010	Canada	Cross-sectional	n = 129; mean (SD) age 15.5 (1.4) 7; range 13-17 7	Cerebral palsy	Child or parent	AQoL; HUI-3
Tilford et al^26^	2012	US	Cross-sectional	n = 150; mean (SD) age 8.6 (3.3) 7; range 4-17 7; 14.7% female	Autism spectrum disorder	Caregiver	HUI-3; QWB
Petrou et al^27^	2013	UK; Ireland	Cross-sectional	n = 79; median age 10.9 7; range 10.1-11.1 7; 44.3% female	Neurodevelopmental disability	Parent	HUI-2; HUI-3
Kulpeng et al^28^	2013	Thailand	Cross-sectional	n = 173; mean (SD) age 10 (3) 7; range 5-14 7; 38% female	Hearing loss; "mild mental retardation"; "severe mental retardation"; "mental retardation combined with epilepsy"	Caregiver or caregiver/child pair	EQ-5D; HUI-2; HUI-3
Burström et al^29^	2014	Sweden	Cross-sectional	n = 71; mean (SD) age 12.0 (3.1) 7; range 7-17 7; 60.6% female	Arthrogryposis multiple congenital; myelomeningocele; cerebral palsy; orthopedic lower-limb deformities; juvenile idiopathic arthritis; achondroplasia	Child	EQ-5D-Y
Domellöf et al^30^	2014	Sweden	Cross-sectional	n = 175; mean age 11.7 7; range 7-17 7; 32.6% female	Intellectual disabilities; autism spectrum disorders; movement disorders; hearing disabilities	Child or parent	EQ-5D-Y
Chevreul et al^31^	2015	France	Cross-sectional	n = 53; mean (SD) age 10.3 (4.3) 7; 11.3% female	Fragile X syndrome	Caregiver	EQ-5D-5 L
Chevreul et al^32^	2016	France	Cross-sectional	n = 25; mean (SD) age 6.8 (4.9) 7; 52% female	Pradar-Willi syndrome	Parent	EQ-5D-5 L
Landfeldt et al^33^	2016	Germany; Italy; UK; US	Cross-sectional	n = 770; ≥5 7; 100% male	Duchenne muscular dystrophy	Child or parent	HUI
Hind et al^34^	2017	UK	RCT	n = 12; mean (SD) age 8.6 (1.7) 7; range 7-13 7; 100% male	Duchenne muscular dystrophy	Child	CHU-9D
Ramanan et al^35^	2019	UK	RCT	n = 90; mean (SD) 8.90 (3.9) 7; 78% female	Uveitis associated with juvenile idiopathic arthritis	Parent or caregiver	HUI-3
Le et al^36^	2020	Australia	Cross-sectional	(a) Children with typical (n = 886) and low language abilities (n = 126); n = 1012; mean (SD) age 4.2 (0.1) 7; 46% female (b) Children with congenital hearing loss; n = 108; mean (SD) age 5.3 (0.8) 7; 55% females	(a) Typical and low language abilities (b) Congenital hearing loss	Child or parent	HUI-3; PedsQL
Kirkham et al^37^	2020	Germany; Italy; Spain; UK	Cross-sectional	n = 286; mean (SD) age 8.8 (3.8) 7; range 3-16 7	Learning disability associated with epilepsy	Clinician, parent and child	EQ-5D-3 L
Nair et al^38^	2020	India	Cross-sectional	(a) Patients with Usher syndrome: patients; n = 27; mean age 2.9 7; range 11 mo to 4.7 7 (b) Patients without Usher syndrome: n = 30; mean age 4.1 7; range 1.8-6 7	Usher syndrome (hearing and vision loss)	Not clear	HUI-3
Tonmukayakul et al^39^	2020	Australia	RCT	n = 76; mean (SD) age 9:7 (3:0) 7; range 6-15 7; 53% female	Cerebral palsy	Parent or caregiver	CHU-9D
Liu et al^40^	2021	New Zealand	Cross-sectional	n = 127; corrected age 7 7; 47% female	Children born <30 weeks' gestation or <1500 g birth weight with NDI categorized as mild and severe NDI cases: - Mild NDI is determined by certain criteria related to cognitive and motor skills - Severe NDI encompassed a broader range of criteria, including factors like very low IQ, significant motor challenges, cerebral palsy, hearing impairment requiring aids, or severe visual impairment.	Caregiver	CHQ-PF50; HUI-2
Randell et al^41^	2022	UK	RCT	n = 138; mean (SD) age 7.87 (1.73) 7; 21% female	Autism	Caregiver	EQ-5D-5 L
van Westrhenen et al^42^	2023	Netherlands	RCT	n = 53; mean (SD) age 9.7 (±3.6) 7; range 4-16; 45% females	Learning disability associated with epilepsy	Caregiver	EQ-5D-5 L
Khan et al^43^	2023	US; Israel	RCT	n = 21; mean (SD) age 15 (1.3) 7; range 13-17; 48% females	Angelman syndrome	Child or parent	EQ-5D; EQ-5D VAS
Da Costa et al^44^	2023	Brazil	Cross-sectional	n = 86; range 5-12; a) Developmental disabilities (n = 52); mean (SD) age 7.5 (±2) 7; 33% females; b) Typical development (n = 34); mean (SD) age 7.1 (±2.1) 7; 50% females	Cerebral palsy; Down syndrome, Myelomeningocele; Congenital malformations; and autism, among others	Caregiver	PedsQL
Bukhari and Zawawi^46^	2024	Saudi Arabia	Cross-sectional	n = 79 ages 13-18 7	Hearing loss	Children	HEAR-QL
Blackmore et al^48^	2024	Australia	Cross-sectional	n = 28 (30 caregivers reporting on 28 children) ages 8-22 7	Intellectual disability	Caregivers	EQ-5D-Y-5L
Downs et al^47^	2024	Australia	Cross-sectional	n = 234 ages 4-18 7	Intellectual disability	Caregivers	EQ-5D-Y-5L

*NDI*, neurodevelopmental impairments; *RCT*, randomized controlled trial.

### Assessment of Health-Related Quality of Life (HRQoL) Utility Values

Among the 31 studies included, response rates ranged from 40% to 100%. The reasons for nonresponse were not reported for most studies. When provided, the reasons cited included participants' limited ability to self-report, refusal to participate, inaccessibility of participants, and failure to return questionnaires. The review identified a diverse range of measures used to assess HSUVs in CAD (Table II). Only 2 studies used direct methods (TTO, VAS, SG),^18^^,^^23^ in contrast to an array of 13 different indirect methods used across the other studies. Notably, the most frequently used indirect method was Health Utilities Index 3 (HUI-3; 11 studies),^20^^,^^22^^,^^24^, ^25^, ^26^, ^27^, ^28^^,^^35^^,^^36^^,^^38^^,^^45^ followed by Health Utilities Index 2 (HUI-2; 4 studies),^19^^,^^27^^,^^28^^,^^40^ EuroQol 5 Dimensions 5 Level (EQ-5D-5 L; 6 studies),^31^^,^^32^^,^^41^^,^^42^^,^^46^^,^^47^ and EQ-5D (3 studies).^21^^,^^28^^,^^43^ Pediatric Quality of Life Inventory (PedsQL), EuroQol-5 Dimension Youth (EQ-5D-Y), QWB, CHU-9D, and HUI were each used in 2 studies.^22^^,^^26^^,^^29^^,^^30^^,^^33^^,^^34^^,^^36^^,^^39^^,^^44^^,^^46^ The remaining measures, including Hearing Environments and Reflection of Quality-of-Life questionnaire, EuroQol 5 Dimensions 3 Level (EQ-5D-3 L), EQ-5D VAS, Child Health Questionnaire-Parent Form 50 (CHQ-PF50), and AQoL, were each employed once.^25^^,^^37^^,^^40^^,^^43^ Further details on the methods used to derive and score the HSUVs from these measures are available in Tables II and III.

**Table II:** Methods used for obtaining utility values

Types	Number of studies, No.	Included authors using this method	Year
Direct methods			
TTO	2	Carroll et al^23^	2009
		Cheng et al^18^	2000
SG	1	Carroll et al^23^	2009
VAS	1	Cheng et al^18^	2000
Indirect methods			
HUI-3	11	Barton et al^20^	2006
		Rosenbaum et al^45^	2007
		Smith-Olinde et al^22^	2008
		Petrou et al^24^	2009
		Young et al^25^	2010
		Tilford et al^26^	2012
		Petrou et al^27^	2013
		Kulpeng et al^28^	2013
		Ramanan et al^35^	2019
		Le et al^36^	2020
		Nair et al^38^	2020
HUI-2	4	Tilford et al^19^	2005
		Petrou et al^27^	2013
		Kulpeng et al^28^	2013
		Liu et al^40^	2021
EQ-5D-5 L	6	Chevreul et al^31^	2015
		Chevreul et al^32^	2016
		Randell et al^41^	2022
		van Westrhenen et al^42^	2023
		Downs et al^47^	2024
		Blackmore et al^48^	2024
EQ-5D	3	Sach et al^21^	2007
		Kulpeng et al^28^	2013
		Khan et al^43^	2023
PedsQL	2	Le et al^36^	2020
		Da Costa et al^44^	2023
EQ-5D-Y	2	Burström et al^29^	2014
		Domellöf et al^30^	2014
QWB	2	Smith-Olinde et al^22^	2008
		Tilford et al^26^	2012
CHU-9D	2	Hind et al^34^	2017
		Tonmukayakul et al^39^	2020
HUI	2	Cheng et al^18^	2000
		Landfeldt et al^33^	2016
EQ-5D-3 L	1	Kirkham et al^37^	2020
EQ-5D VAS	1	Khan et al^43^	2023
CHQ-PF50	1	Liu et al^40^	2021
AQoL	1	Young et al^25^	2010
HEAR-QL	1	Bukhari and Zawawi^46^	2024

**Table III:** Scoring method used to obtain utility values

Measure	Method used	Authors	Year	Included authors using this method	Year
HUI-2	Canadian scoring function	Furlong et al^49^	2002	Kulpeng et al^28^	2013
	UK adult population	McCabe et al^50^	2005	Petrou et al^27^	2013
	Algorithms for assigning preference scores developed using community samples	Not provided		Tilford et al^19^	2005
	Normative reference population	HealthActCHQ	2013	Liu et al^40^	2021
HUI-3	Canadian general population preferences for health status	Feeny et al^51^	2002	Petrou et al^24^	2009
				Petrou et al^27^	2013
				Barton et al^20^	2006
				Tilford et al^26^	2012
	Unclear	Feeny et al	1996	Young et al^25^	2010
	Canadian general population preferences for health status	Furlong et al	1998	Rosenbaum et al^45^	2007
				Ramanan et al^35^	2019
	Canadian scoring function	Furlong et al^49^	2002	Kulpeng et al^28^	2013
	Algorithms for assigning preference scores developed using community samples	Not provided		Smith-Olinde et al^22^	2008
	Canadian population preference weights	Drummond et al	2001	Le et al^36^	2020
	Not stated	Not provided		Nair et al^38^	2020
HUI	General adult population	Not provided		Cheng et al^18^	2000
	General public	Horsman et al	2003	Landfeldt et al^33^	2016
AQoL	Not stated	Hawthorne et al	2001	Young et al^25^	2010
		Hawthorne et al	1999		
EQ-5D	Not stated	Not provided		Sach et al^21^	2007
	Thai algorithm	Tongsiri et al	2011	Kulpeng et al^28^	2013
	Not stated	Not provided		van Westrhenen et al^42^	2023
EQ-5D-3 L; EQ-5D-Y	UK-specific weightings	Dolan et al	1995	Kirkham et al^37^	2020
EQ-5D-5 L	European adult population	Van Hout et al	2012	Chevreul et al^31^	2015
				Chevreul et al^32^	2016
	Not stated	Not provided		Randell et al^2^	2022
				Downs et al^47^	2024
				Blackmore et al^48^	2024
EQ-5D; EQ-5D VAS	US population preference weights	Szende et al	2014	Khan et al^43^	2023
PedsQL	Canadian population preference weights	Drummond et al	2001	Le et al^36^	2020
	Not stated	Not provided		Da Costa et al^44^	2023
QWB	Preference weights derived from a representative community sample	Not provided		Smith-Olinde et al^22^	2008
				Tilford et al^26^	2012
CHU-9D	Australian adolescent population-specific scoring algorithm	Ratcliffe et al	2001	Tonmukayakul et al^39^	2020
	Not stated	Not provided		Hind et al^34^	2017
HEAR-QL	Not stated	Not stated		Bukhari & Zawawi^46^	2024

In specific populations, such as children with sensory impairment (12 studies), 2 studies employed direct methods (TTO, VAS, and SG),^18^^,^^23^ whereas the most frequently used indirect method was the HUI-3 (7 studies).^20^^,^^22^^,^^24^^,^^28^^,^^35^^,^^36^^,^^38^ The EQ-5D and HUI-2 were each used in 3 studies.^21^^,^^28^^,^^48^ Other methods, including CHQ-PF50, EQ-5D-Y, HUI, PedsQL, and QWB, also were employed.^22^^,^^30^^,^^36^^,^^40^^,^^46^ In studies focusing on children with speech or language disorders (3 studies), HSUVs were measured using the HUI-3 in 2 studies,^24^^,^^36^ whereas other methods such as CHQ-PF50, HUI-2, and PedsQL also were employed.^24^^,^^36^^,^^40^ For children with primary physical impairments, such as cerebral palsy and spina bifida (9 studies),^19^^,^^23^, ^24^, ^25^^,^^29^^,^^33^^,^^34^^,^^39^^,^^45^ only one study employed direct methods (SG and TTO).^23^ HUI-3 was used in 3 studies,^24^^,^^25^^,^^45^ and CHU-9D was used in 2 studies.^34^^,^^39^ Other methods, such as AQoL, EQ-5D-Y, HUI, and HUI-2 were also employed.^19^^,^^25^^,^^29^^,^^33^ For children and adolescents with autism spectrum disorder (5 studies), 2 studies employed the HUI-3,^24^^,^^26^ whereas the remaining studies used a variety of measures including EQ-5D-Y, EQ-5D-5 L, PedsQL, and QWB.^26^^,^^30^^,^^41^^,^^44^ For children with intellectual impairment (4 studies), HSUVs were assessed using diverse direct and indirect measures, including SG, TTO, HUI-3, EQ-5D-3 L, and EQ-5D-5 L.^23^^,^^24^^,^^37^^,^^42^^,^^46^^,^^47^ For children with developmental disabilities (8 studies), 3 studies used the HUI-3,^24^^,^^26^^,^^27^ 2 applied the HUI-2,^27^^,^^40^ and 2 incorporated the EQ-5D-5 L.^31^^,^^32^ The remaining studies employed a variety of measures, including other variants of the EQ-5D (such as EQ-5D and EQ-5D VAS),^43^ as well as PedsQL, QWB, CHQ-PF50,^26^^,^^40^^,^^43^ and Hearing Environments and Reflection of Quality-of-Life questionnaire.^48^

Despite this diversity, some studies did not reference the scoring algorithm used or describe the method used in detail. Those that did provide this information often used an algorithm on the basis of the preferences of the general population, as this is typical for utility instruments like the HUI and EQ-5D. These algorithms reflect public preferences and, in some cases, may vary by country-specific value sets for use in CUAs. Furthermore, some studies employed different versions of the same generic measure (eg, EQ-5D-3 L, EQ-5D-5 L, and EQ-5D VAS), potentially affecting result comparability.

### Administration Methods

A parent or caregiver was the only respondent in the majority of studies (n = 19, 61%).^18^, ^19^, ^20^, ^21^, ^22^, ^23^, ^24^^,^^26^^,^^27^^,^^31^^,^^32^^,^^35^^,^^39^, ^40^, ^41^, ^42^^,^^44^^,^^46^^,^^47^ In 6 studies, the respondent was either a child or parent.^25^^,^^30^^,^^33^^,^^36^^,^^43^^,^^45^ The child was the respondent in only 3 studies.^29^^,^^34^^,^^48^ The respondent was not reported in 1 study.^38^ One study included a combination of clinicians, parents, and/or children,^37^ whereas another study involved a caregiver/child pair as the respondent.^28^

In 10 studies, researchers obtained HSUVs through interviews.^23^, ^24^, ^25^^,^^30^^,^^39^^,^^41^^,^^42^^,^^45^, ^46^, ^48^ Postal questionnaires were employed in 7 studies,^24^^,^^26^^,^^28^^,^^32^^,^^33^^,^^46^^,^^48^ whereas online questionnaires were employed in 6.^21^^,^^22^^,^^29^^,^^37^^,^^43^^,^^47^ Three studies used self-administered questionnaires completed in clinical settings.^27^^,^^31^^,^^34^ In 1 study, participants had the option to complete the questionnaire either in a clinical setting or via postal delivery.^20^ The remaining studies (n = 7, 25%) did not specify the methodology used to elicit HSUVs.

The type of missing data varied across different measures. However, these missing data could be attributed to factors such as participant dropout, loss to follow-up, or incomplete responses by the participants. For HUI-3 and HUI-2, missing data ranged from 0% to 20.7%^20^^,^^25^^,^^26^^,^^35^^,^^45^ and 0% to 18.4%,^19^^,^^25^ respectively. The EQ-5D had the lowest rate of missing data (0% to 1%),^21^^,^^25^ whereas the EQ-5D-Y had slightly greater rates of missing data (2.8%-4.2%).^29^^,^^30^ For EQ-5D-5 L missing data ranged between 17% and 28.3%.^32^^,^^42^ The CHU-9D had the greatest rate of missing data, up to 43%, attributed to self-reporting limitations.^36^ One study reported no missing data for the QWB.^26^

### Psychometric Properties of the Methods Used to Obtain Health State Utility Values

Thirteen studies reported the validity of instruments to obtain health service utility values. Table IV details the construct validity (convergent and/or known-groups validity) of HUI-3, HUI-2, HUI (mark not stated), AQoL, QWB, EQ-5D, EQ-5D-5 L, EQ-5D-Y, and CHU-9D among CAD. Eight studies evaluated the construct validity of the HUI-3,^20^^,^^22^^,^^25^, ^26^, ^27^, ^28^^,^^36^^,^^45^ and 3 evaluated the construct validity of the HUI-2.^19^^,^^27^^,^^28^ The QWB^22^^,^^26^ and EQ-5D-Y^29^^,^^30^ were each examined in 2 studies, whereas 1 study assessed the EQ-5D,^25^ AQoL,^25^ HUI (mark not stated),^33^ and CHU-9D.^39^ There was some evidence of construct validity for all generic measures. HSUVs from HUI-3, HUI-2, AQoL, QWB, and EQ-5D were significantly correlated with HRQoL on other generic measures.^25^^,^^28^^,^^36^^,^^45^ However, weak correlations were observed between HUI-3 and a condition-specific measure for cerebral palsy,^25^ as well as between CHU-9D utility scores and the Cerebral Palsy Quality of Life Questionnaire.^36^ Weak correlations were also noted between HUI-3 and PedsQL domains for children with language and/or hearing disabilities.^44^ Further analysis indicated significant correlations between HSUVs from HUI-3, HUI, AQoL, and QWB and severity in children with developmental disability, sensory impairment, and physical impairment,^20^^,^^21^^,^^24^, ^25^, ^26^ except for those with autism.^32^ No studies were identified that reported the content, criterion validity or responsiveness of the instruments in CAD. One study^52^ reported reliability coefficients of 0.94 for HUI-2, 0.86 for EQ-5D and 0.87 for HUI-3 in CAD.

**Table IV:** Psychometric properties of generic measures

Measures	Authors	Years	Validity∗		Missing data∗
			Convergent and/or known-groups validity	Construct	
HUI-3	Young et al^25^	2010	Strong correlation with AQoL, Moderate correlation with EQ-5D	Utility strongly associated with severity of motor impairment	No missing data
	Kulpeng et al^28^	2013	Strong correlation with HUI-2, Moderate correlation with EQ-5D	–	–
	Smith-Olinde et al^22^	2008	No difference between QWB and HUI-3 utility scores	Utilities declined with increasing hearing loss and increasing severity of hearing loss for children without cochlear implant	–
	Rosenbaum et al^45^	2007	Weak correlation with the quality-of-life Instrument for People with Developmental Disabilities	Utility strongly associated with severity of motor impairment	2%participants had missing data for ≥1 domains
	Le et al^36^	2020	Moderate correlation between HUI-3 and PedsQL overall scores in the full general population sample, as well as in children with low language, but not in children with congenital hearing loss^36^ Low correlations observed between each of the HUI-3 and the PedsQL domains in the general population, as well as in the groups of children with low language or congenital hearing loss	Children with low language had lower HRQoL than their peers with typical language, as evidenced by the HUI-3 scores (6% difference in the general population and 19% and 30% differences in children with congenital hearing loss) The PedsQL scores did not show significant HRQoL differences between children with and without low language in either cohort	
	Petrou et al^27^	2013	–	Difference in utility between children with and without neurodevelopmental disability^27^	
	Tilford et al^26^	2012	–	Utility not associated with Autism Diagnostic Observation Schedule calibrated severity score^26^	2.7% participants had missing utility value^26^
	Barton et al^20^	2006	–	–	20.7% participants had missing data for ≥1 domains
	Young et al^25^	2010	–	–	No missing data^25^
HUI-2	Kulpeng et al^28^	2013	Strong correlation with HUI-3 Moderate correlation with EQ-5D	–	–
	Petrou et al^27^	2013	–	Difference in utility between children with and without neurodevelopmental disability	–
	Tilford et al^19^	2005	–	Difference in utility between children with and without spina bifida Utility declined with increasing severity of lesion	18.4% of participants had missing data for ≥1 domains
	Young et al^25^	2010	–	–	No missing data^25^
HUI (mark not stated)	Landfeldt et al^33^	2016	–	Utility associated with disease progression and caregivers' rating of the child's current health^33^	–
AQoL	Young et al^25^	2010	Strong correlation with HUI-3	Utility moderately associated with severity of motor impairment	–
QWB	Smith-Olinde et al^22^	2008	No difference between QWB and HUI-3 utility scores	Utility declined with increasing severity of hearing loss for children without cochlear implant	–
	Tilford et al^26^	2012	–	Utility not associated with Autism Diagnostic Observation Schedule calibrated severity score	No participants had missing utility value
EQ-5D	Sach et al^21^	2007	–	–	0.5% participants missing data
EQ-5D-5 L	Chevreul et al^32^	2016	–	–	17% participants missing data
	van Westrhenen et al^42^	2023	–	–	24.5% participants had missing data at baseline and 28.3% at the follow-up
	Downs et al^47^	2024	EQ-5D-Y-5L is suitable for assessing HRQoL in children with intellectual disability, with limitations in EQ-VAS stability and some dimensions.	Strong validity for mobility, self-care, and pain dimensions; fair to moderate test-retest reliability; variable EQ-VAS performance	Less than 1% missing data
	Blackmore et al^48^	2024	EQ-5D-Y-5L shows basic validity but lacks comprehensiveness for HRQoL in children with intellectual disability; further adaptation recommended.	–	Missing data reported as ‘minimal’
EQ-5D-Y	Burström et al^29^	2014	“Feeling worried, sad or unhappy” dimension negatively moderately associated with psychological well-being dimension in KIDSCREEN “Mobility” dimension not associated with physical well-being dimension of KIDSCREEN Moderate correlations between Visual Analogue Scale and KIDSCREEN HRQoL index, self-rated general health item and life satisfaction ladder	“Some” or “a lot of” problems on any dimension was reported by 82.9% of children with disability, compared with 36.6% of children in general population	4.2% of participants had missing values for ≥1 dimension
	Domellöf et al^30^	2014	–	Between diagnostic group differences for all dimensions	2.8% of participants had missing values for ≥1 dimension
CHU-9D	Tonmukayakul et al^39^	2020	Weak correlation between the overall Cerebral Palsy Quality of Life Questionnaire-Child score and the CHU-9D utility scores At the domain level, the participation and emotional wellbeing domains showed a moderate positive correlation with the CHU-9D scores, while the feelings and social wellbeing domains demonstrated strong positive correlations No significant correlations were found between the CHU-9D scores and the access to service domain The pain domain had a negative but non-significant correlation with the CHU-9D scores	Greater upper-limb impairment was associated with lower HRQoL. However, the relationship was weak and may be due to the fact that more than half of the participants had mild upper-limb impairment	Up to 43% of participants had missing data as many had limited ability to self-report
HEAR-QL	Bukhari and Zawawi^46^	2024	Discriminatie validity established with the HEAR-QL tool	Normal hearing group had the highest QoL scores, followed by the CI group, with the untreated hearing loss group scoring the lowest.	Not reported

∗ Some of the studies did not report details for convergent validity, construct validity, or missing data for their reported HSUVs.

### Summary Statistics of Reported Health State Utility Values

Tables V and VI report the HSUVs of children and adolescents by disability type. However, in 4 studies, HSUVs were not available.^23^^,^^27^^,^^28^^,^^30^ There was variation in reported HSUVs across measures, even for children and adolescents with the same condition and severity. It should be noted that these ranges may reflect differences in study design, populations, or the instruments used, making comparisons across studies potentially misleading. For example, the range of (−0.13 to 0.95; Table V) for individuals with primary physical impairments includes data from a cross-sectional study of children with cerebral palsy and a randomized controlled trial involving children with Duchenne muscular dystrophy.^25^^,^^34^ For those with sensory impairment, mean values ranged from 0.25 to 0.99 (Table V).^18^^,^^35^ Among those with speech or language disorders, one study reported mean values ranging from 0.72 to 0.85 (Table V),^36^ whereas another study documented a median of 0.53 (Table V).^24^^,^^25^^,^^34^ Similarly, mean values for children and adolescents with autism spectrum disorders spanned from 0.58 to 0.84 (Table VI).^26^^,^^41^ For children and adolescents with intellectual impairment, mean values fell within the range of 0.5-0.9 (Table VI).^37^^,^^42^ Lastly, for individuals with developmental disabilities, mean values ranged from 0.42 to 0.76 (Table VI).^31^

**Table V:** Health state utility values for children and adolescents with sensory impairment, speech or language disorders, primary physical disability

Authors	Year	Condition	Method	Sample size, No.∗	Mean (SD)∗	Median (IQR)∗
Sensory impairment						
Carroll et al^23^	2009	Mild hearing loss	SG	–	0.92 (0.16)	0.99
		Mild hearing loss	TTO	–	0.93 (0.17)	0.99
Smith-Olinde et al^22^	2008	Mild/moderate hearing loss	HUI-3	22	0.71 (0.18)	–
		Mild/moderate hearing loss	QWB	22	0.65 (0.12)	–
Carroll et al^23^	2009	Moderate hearing loss	SG	–	0.91 (0.18)	0.99
		Moderate hearing loss	TTO	–	0.92 (0.18)	0.99
Barton et al^20^	2006	Moderate hearing loss	HUI-3	260	0.68	–
Smith-Olinde et al^22^	2008	Moderate/severe hearing loss	HUI-3	34	0.62 (0.22)	–
		Moderate/severe hearing loss	QWB	34	0.59 (0.11)	–
Carroll et al^23^	2009	Severe hearing loss	SG	–	0.86 (0.19)	0.94
		Severe hearing loss	TTO	-	0.86 (0.20)	0.94
Barton et al^20^	2006	Severe hearing loss	HUI-3	464	0.62	–
		Profound hearing loss (AHL 96-105 dB)	HUI-3	259	0.50	–
		Profound hearing loss (AHL >105 dB)	HUI-3	290	0.35	–
Smith-Olinde et al^22^	2008	Severe/profound hearing loss (no implant)	HUI-3	19	0.54 (0.22)	–
		Severe/profound hearing loss (no implant)	QWB	19	0.55 (0.07)	–
Cheng et al^18^	2000	Profound deafness (no implant)	VAS	78	0.59	–
		Profound deafness (no implant)	TTO	40	0.75	–
		Profound deafness (no implant)	HUI	22	0.25	–
Smith-Olinde et al^22^	2008	Hearing loss	HUI-3	103	0.62 (0.20)	–
		Hearing loss	QWB	103	0.60 (0.11)	–
Petrou et al^24^	2009	Deafness	HUI-3	104	–	0.41
Smith-Olinde et al^22^	2008	Severe/profound hearing loss (implant)	HUI-3	28	0.61 (0.16)	–
		Severe/profound hearing loss (implant)	QWB	28	0.61 (0.09)	–
Barton et al^20^	2006	Hearing loss with implant	HUI-3	403	0.58	10db
Cheng et al^18^	2000	Profound deafness (implant)	VAS	78	0.86	50 dbSPL
		Profound deafness (implant)	TTO	40	0.97	22.5 SPL
		Profound deafness (implant)	HUI	40	0.64	–
Sach et al^21^	2007	Hearing loss (implant)	EQ-5D	215	0.88 (0.17)	–
Petrou et al^24^	2009	Deafness with other impairments	HUI-3	15	–	0.40
Carroll et al^23^	2009	Mild bilateral vision loss	SG	–	0.89 (0.18)	0.97
		Mild bilateral vision loss	TTO	–	0.91 (0.19)	0.99
		Moderate bilateral vision loss	SG	–	0.85 (0.22)	0.94
		Moderate bilateral vision loss	TTO	–	0.86 (0.21)	0.94
		Severe bilateral vision loss	SG	–	0.81 (0.22)	0.89
		Severe bilateral vision loss	TTO	–	0.81 (0.22)	0.89
Le et al^36^	2020	Congenital hearing loss (overall)	HUI-3	108	0.68 (0.26)	0.74 (0.58-0.85)
		Congenital hearing loss (typical language ability)	HUI-3	58	0.79 (0.16)	0.85 (0.73-0.85)
		Congenital hearing loss (low language ability)	HUI-3	43	0.60 (0.24)	0.62 (0.53-0.75)
		Congenital hearing loss (overall)	PedsQL	108	0.75 (0.17)	0.78 (0.65-0.88)
		Congenital hearing loss (typical language ability)	PedsQL	58	0.77 (0.17)	0.79 (0.72-0.89)
		Congenital hearing loss (low language ability)	PedsQL	43	0.72 (0.17)	0.76 (0.62-0.88)
Petrou et al^24^	2009	Vision disorders and blindness	HUI-3	39	–	0.47
Carroll et al^23^	2009	Monocular blindness	SG	–	0.88 (0.17)	0.96
		Monocular blindness	TTO	–	0.89 (0.17)	0.96
Ramanan et al^35^	2019	Mild or moderate uveitis; adalimumab group (baseline)	HUI-3	48	0.83	–
		Mild or moderate uveitis; placebo group (baseline)	HUI-3	21	0.87	–
		Mild or moderate uveitis; adalimumab group (18 mo)	HUI-3	48	0.94	–
		Mild or moderate uveitis; placebo group (18 mo)	HUI-3	21	0.99	–
Nair et al^38^	2020	Patients with Usher syndrome	HUI-3	27	0.43	–
Bukhari and Zawawi^46^	2024	Normal hearing group	HEAR-QL	30		
		uHL hearing group	HEAR-QL	25		
		Moderate hearing loss	HEAR-QL	24		
Speech or language disorders						
Petrou et al^24^	2009	Speech disorders	HUI-3	25	–	0.53
Le et al^36^	2020	Children with low language ability	HUI-3	126	0.85 (0.15)	0.88 (0.76-1)
		Children with low language ability	PedsQL	126	0.72 (0.17)	0.76 (0.62-0.88)
Primary physical disability						
Rosenbaum et al^45^	2007	Cerebral palsy	HUI-3	196	0.42 (0.41)	0.42
Young et al^25^	2010	Cerebral palsy	HUI-3	129	0.30 (0.43)	–
		Cerebral palsy	AQoL	129	0.28 (0.34)	–
Petrou et al^24^	2009	Cerebral palsy	HUI-3	178	–	0.27
Rosenbaum et al^45^	2007	Cerebral palsy (GMFCS level I)	HUI-3	60	0.84 (0.20)	–
Young et al^25^	2010	Cerebral palsy (GMFCS level I)	HUI-3	28	0.67 (0.32)	–
		Cerebral palsy (GMFCS level I)	AQoL	28	0.58 (0.31)	–
Rosenbaum et al^45^	2007	Cerebral palsy (GMFCS level II)	HUI-3	33	0.50 (0.31)	–
Young et al^25^	2010	Cerebral palsy (GMFCS level II)	HUI-3	15	0.59 (0.35)	–
		Cerebral palsy (GMFCS level II)	AQoL	15	0.53 (0.34)	–
Carroll et al^23^	2009	Mild cerebral palsy	SG	–	0.87 (0.20)	0.96
Carroll et al^23^	2009	Mild cerebral palsy	TTO	–	0.88 (0.19)	0.96
		Moderate cerebral palsy	TTO	–	0.76 (0.26)	0.86
Rosenbaum et al^45^	2007	Cerebral palsy (GMFCS level III)	HUI-3	27	0.39 (0.21)	–
Young et al^25^	2010	Cerebral palsy (GMFCS level III)	HUI-3	23	0.43 (0.39)	–
		Cerebral palsy (GMFCS level III)	AQoL	23	0.31 (0.32)	–
Carroll et al^23^	2009	Severe cerebral palsy	SG	–	0.60 (0.28)	0.50
		Severe cerebral palsy	TTO	–	0.55 (0.33)	0.50
Rosenbaum et al^45^	2007	Cerebral palsy (GMFCS level IV)	HUI-3	46	0.16 (0.26)	–
						
Young et al^25^	2010	Cerebral palsy (GMFCS level IV)	HUI-3	32	0.08 (0.25)	–
		Cerebral palsy (GMFCS level IV)	AQoL	32	0.06 (0.12)	–
Rosenbaum et al^45^	2007	Cerebral palsy (GMFCS level V)	HUI-3	30	−0.08 (0.23)	–
						
Young et al^25^	2010	Cerebral palsy (GMFCS level V)	HUI-3	28	−0.13 (0.19)	–
		Cerebral palsy (GMFCS level V)	AQoL	28	0.01 (0.07)	–
		Cerebral palsy (health state C)	HUI-2	–	0.40 (0.11)	0.40
Tonmukayakul et al^39^	2020	Cerebral palsy	CHU-9D	43	0.863 (0.124)	–
		Cerebral palsy (MACS: mild)	CHU-9D	21	0.918 (0.084)	–
		Cerebral palsy (MACS: moderate and severe)	CHU-9D	25	0.825 (0.133)	–
		Cerebral palsy (BFMF: mild)	CHU-9D	30	0.901 (0.816)	–
		Cerebral palsy (BFMF: moderate and severe)	CHU-9D	11	0.813 (0.143)	–
		Cerebral palsy (NHDC: mild)	CHU-9D	20	0.872 (0.118)	–
		Cerebral palsy (NHDC: moderate and severe)	CHU-9D	18	0.858 (0.119)	–
		Cerebral palsy (GMFCS: mild)	CHU-9D	30	0.891 (0.108)	–
		Cerebral palsy (GMFCS: moderate and severe)	CHU-9D	11	0.839 (0.160)	–
Tilford et al^19^	2005	Spina bifida	HUI-2	80	0.55 (0.24)	–
		Spina bifida (sacral lesion, least severe)	HUI-2	34	0.61 (0.26)	–
		Spina bifida (lower lumbar lesion)	HUI-2	27	0.54 (0.19)	–
		Spina bifida (thoracic lesion, most severe)	HUI-2	19	0.45 (0.25)	–
Petrou et al^24^	2009	Unspecified motor disorders	HUI-3	81	0.24	–
Hind et al^34^	2017	Duchenne muscular dystrophy (control group; baseline)	CHU-9D	3	0.92 (0.07)	0.89 (0.87-1.00)
		Duchenne muscular dystrophy (intervention group; baseline)	CHU-9D	8	0.77 (0.23)	0.88 (0.59-0.94)
		Duchenne muscular dystrophy (control group; follow-up)	CHU-9D	1	0.95	0.95
		Duchenne muscular dystrophy (intervention group; follow-up)	CHU-9D	8	0.87 (0.09)	0.87 (0.82-0.95)

*AHL*, average hearing level; *BFMF*, Bimanual Fine Motor Function; *GMFCS*, Gross Motor Function Classification System; *MACS*, Manual Ability Classification System; *NHDC*, Neurological Hand Deformity Classification; *uHL*, untreated hearing loss.

∗ Some of the studies did not report details such as sample size (No.), mean (SD), or median (IQR) for their reported HSUVs.

**Table VI:** Health state utility values for children and adolescents with autism spectrum disorders, intellectual impairments, and developmental disabilities

Authors	Year	Condition	Method	Sample size, No.∗	Mean (SD)∗	Median (IQR)∗
Autism spectrum disorders						
Petrou et al^24^	2009	Autism spectrum disorders	HUI-3	105	–	0.41
Tilford et al^26^	2012	Autism spectrum disorder	HUI-3	146	0.66 (0.23)	–
		Autistic disorder	HUI-3	110	0.64 (0.23)	–
		Asperger's disorder	HUI-3	13	0.79 (0.16)	–
		Autism spectrum disorder	QWB	150	0.59 (0.16)	–
		Autistic disorder	QWB	114	0.58 (0.16)	–
		Asperger disorder	QWB	13	0.62 (0.15)	–
Randell et al^41^	2022	Autism and sensory processing difficulties (control group; baseline)	EQ-5D-5 L	69	0.84	0.74-0.88
		Autism and sensory processing difficulties (control group; 6 mo)	EQ-5D-5 L	35	0.84	0.73-0.88
		Autism and sensory processing difficulties (control group; 12 mo)	EQ-5D-5 L	24	0.78	0.69-0.88
		Autism and sensory processing difficulties (intervention group; baseline)	EQ-5D-5 L	69	0.77	0.72-0.88
		Autism and sensory processing difficulties (intervention group; 6 mo)	EQ-5D-5 L	48	0.77	0.74-0.88
		Autism and sensory processing difficulties (intervention group; 12 mo)	EQ-5D-5 L	36	0.84	0.71-0.88
Downs et al^47^	2024	Intellectual disability, including autism spectrum disorder, cerebral palsy, Down syndrome, and other genetic conditions	EQ-5D-5 L	234	–	–
Blackmore et al^48^	2024	Intellectual disability, including autism spectrum disorder, Down syndrome, and cerebral palsy	EQ-5D-5 L	28	–	–
Intellectual impairments						
Petrou et al^24^	2009	Learning disabilities	HUI-3	251	–	0.40
Carroll et al^23^	2009	“Mild mental retardation”	SG	–	0.84 (0.20)	0.91
		“Mild mental retardation”	TTO	–	0.83 (0.23)	0.93
		“Mild mental retardation”	SG	–	0.79 (0.22)	0.86
		“Mild mental retardation”	TTO	–	0.79 (0.23)	0.87
		“Severe mental retardation”	SG	–	0.59 (0.27)	0.50
		“Severe mental retardation”	TTO	–	0.51 (0.32)	0.50
Petrou et al^24^	2009	Severe learning disabilities/global developmental delay	HUI-3	118	–	0.39
		Down's syndrome	HUI-3	155	–	0.34
Kirkham et al^37^	2020	Learning disabilities—reported by clinician	EQ-5D-3 L proxy version	279	0.52 (0.41)	0.71 (−0.594, 1)
		Learning disabilities—reported by parent	EQ-5D-3 L proxy version	277	0.51 (0.39)	0.69 (−0.594, 1)
		Learning disabilities—reported by patient	EQ-5D-3 L and EQ-5D-Y	85	0.74 (0.29)	0.81 (−0.166, 1)
		Learning disabilities—reported by patient aged 7-12 7	EQ-5D-Y	58	0.72 (0.30)	0.80 (−0.166, 1)
		Learning disabilities–reported by patient aged 13-16 y	EQ-5D-3 L	27	0.78 (0.27)	(0.151, 1)
van Westrhenen et al^42^	2023	Learning disabilities associated with Epilepsy (baseline)	EQ-5D-5 L	53	0.9	–
		Learning disabilities associated with Epilepsy (follow-up)	EQ-5D-5 L	53	0.9	–
Developmental disabilities						
Petrou et al^24^	2009	Learning and physical disabilities	HUI-3	81	–	0.12
Da Costa et al^44^	2023	Children with developmental disabilities	PedsQL	52	–	0.538 (0.174-0.81)
Khan et al^43^	2023	Adolescents with Angelman syndrome	EQ-5D	21	0.42 (0.20)	0.49 (0.06-0.74)
		Adolescents with Angelman syndrome	EQ-5D VAS	21	0.82 (0.16)	0.89 (0.49- 1)
Chevreul et al^31^	2015	Fragile X syndrome	EQ-5D-5 L	53	0.46 (0.23)	–
Chevreul et al^32^	2016	Pradar-Willi syndrome	EQ-5D-5 L	10	0.51 (0.33)	–
Tilford et al^26^	2012	Pervasive developmental disorder	HUI-3	23	0.70 (0.24)	–
		Pervasive developmental disorder	QWB	23	0.62 (0.18)	–
Petrou et al^27^	2013	Neurodevelopmental disability	HUI-2	79	0.76	–
		Neurodevelopmental disability	HUI-3	79	0.65	–
Liu et al^40^	2021	Neurodevelopmental impairment	HUI-2	60	Not provided	0.92 (0.83, 0.96)
		Neurodevelopmental impairment	CHQ-PF50 - Physical summary score	60	48.0 (13.1)	–
		Neurodevelopmental impairment	CHQ-PF50 - Psychosocial summary score	60	45.9 (9.5)	–

∗ Some of the studies did not report details such as sample size (No.), mean (SD), or median (IQR) for their reported HSUVs.

## Discussion

This systematic review synthesizes literature on measures used to assess HSUVs in CAD and provides summary statistics for the reported HSUVs. A wide range of measures were used, including both direct methods such as TTO, SG, and VAS, and indirect methods like EQ-5D and HUI, among others. Administration methods varied across studies, from interviews to postal or online questionnaires, with some studies lacking specification, highlighting a lack of standardization in data collection. Furthermore, the wide variation in HSUVs across different types of impairments suggests potential challenges in drawing accurate conclusions from CUA of CAD and may result in inadequately informed decisions regarding healthcare interventions.

The validity of HSUVs can be impacted by factors such as who administers the instrument, their training, the mode of administration, and the respondent.^47^ The majority of included studies identified the respondent, with only three studies using children as exclusive respondents,^29^^,^^34^^,^^48^ and one involved a caregiver-child dyad.^28^ Heavy reliance on proxies to elicit HSUVs in CAD also raises concerns about the accuracy and generalizability of the resulting estimates. In fact, more than one-half of the studies (61%, n = 19)^18^, ^19^, ^20^, ^21^, ^22^, ^23^, ^24^^,^^26^^,^^27^^,^^31^^,^^32^^,^^35^^,^^39^, ^40^, ^41^, ^42^^,^^44^^,^^46^^,^^47^ used a parent or caregiver as the proxy respondent despite evidence that children and adolescents can complete utility assessments.^53^ Furthermore, only 4 studies reported training assessors,^23^^,^^25^^,^^31^^,^^45^ leaving unanswered questions about the respondents' comprehension of the instrument and the accuracy of their responses.

Our findings indicate a tendency to use adult-specific methods to obtain HSUVs among CAD, aligning with patterns observed in previous published literature.^23^^,^^54^ None of the studies used direct preference elicitation from CAD. In fact, the majority of studies employed generic measures, with only 4 using instruments that have been validated for use in children and adolescents (CHU-9D and EQ-5D-Y).^29^^,^^30^^,^^34^^,^^39^ Although the lack of appropriate tools in the field may justify the use of nonchild and adolescent specific instruments, their validity and generalizability in eliciting HSUVs is uncertain. Moreover, the scoring methods employed to derive HSUVs were also a matter of concern. Eight studies incorporated preferences obtained from either the general population or adults,^20^, ^21^, ^22^, ^23^^,^^25^^,^^33^^,^^42^^,^^44^ which may not accurately reflect the preferences/values of CAD. This variation significantly hinders the generalizability of the findings. Of significant concern too is the absence or inadequate use of child-specific preferences. For instance, the techniques used to derive utility scores were not reported in a study that employed the child-specific CHU-9D.^38^ This, in turn, can potentially compromise evidence underpinning the cost-effectiveness of healthcare interventions and resource allocation decisions. However, there is a lack of consensus on optimal approaches for eliciting and measuring HSUVs, which may explain the variability across studies.^52^ It is therefore imperative to establish a consensus to ensure reliable and comparable outcomes across studies of CAD.

The psychometric properties of the generic measures used in CAD were mixed, with some measures showing good construct validity for specific diagnoses, whereas others did not. Consistent with recent reviews on generic childhood multi-attribute utility instruments, evidence for psychometric instruments in CAD is primarily available for the HUI-3, with known-groups validity as the most frequently assessed property. Similar gaps in evidence were observed for instrument reliability in CAD. Notably, no psychometric data were found for the Sixteen-/Seventeen-dimensional (16D/17D)-HRQoL instruments, Adolescent Health Utility Measure, Child Health Utility 6 Dimensions, Child Health and Social Care Services Pediatric Scale, Infant Quality of Life Instrument, or Teen Assessment of Neurodevelopmental Disabilities Index in the context of CAD.^30^^,^^55^^,^^56^ In terms of construct validity, there was a moderate-to-good agreement observed between EQ-5D, HUI-2, and HUI-3,^23^^,^^31^ but remained unestablished for HUI-3.^32^ Although the latter tool is not child or adolescent specific, the construct validity was established for all HUI instruments (HUI, HUI-2 and HUI-3).^20^^,^^21^^,^^24^, ^25^, ^26^^,^^31^^,^^39^^,^^44^ Furthermore, while convergent validity was established between the QWB and the HUI-3, construct validity was not established when compared with Autism Diagnostic Observation Schedule.^26^ The Autism Diagnostic Observation Schedule was shown to possess good sensitivity, specificity, and predictive ability in children and adolescents, indicating that it is a preferred tool over generic instruments.^55^^,^^57^ Therefore, generic instruments may not be appropriate for assessing disease severity in both general and child/adolescent populations, as they are not designed to capture specific aspects of disease severity. Consequently, it is plausible that the absence of construct validity in AQoL is attributable to the use of child and parent pairs as respondents, rather than relying solely on the children's self-reports. These findings underscore the importance of carefully considering the survey respondent when assessing HSUVs.

Conducting HSUVs relevant to children is acknowledged to be challenging,^58^^,^^59^ since children aged around 7-10 years of age are unable to understand the tasks and make a choice.^60^ This raises questions about the accuracy and appropriateness of using their own preferences in health technology assessments. However, the more pressing issue is the ethical concerns in health state valuation among children. Some topics in the valuation tasks could be sensitive to the adolescent. For example, the state of being dead can pose ethical dilemmas and may cause distress, especially for adolescents.^61^

There is an ongoing debate between the use of proxy (eg, parents or caregivers) vs self-report data in HSUV of children. Although adolescents (aged 11-17) may be able to undertake tasks like pairwise comparisons and best-worst scaling, younger children (under 10) are less capable of completing these tasks independently.^49^^,^^59^ Researchers observed inconsistencies between valuation using adolescent and adult participants. For example, when using the self-reported best-worst scaling approach for the valuation of CHU-9D states, the worst choices were far less consistent than the best choices among adolescents compared with adults.^50^ Researchers recommended that HSUVs of children should be considered valid in the tailored methods that account for children's developmental stages, an appropriate group for preferences (eg, children, adolescents, or adults) for aligning with the broader discussions on resource allocation and health equity for younger populations.^50^

This review underscored the potential ableism inherent in the current HSUV measures, which may equate lower functional capacity with diminished quality of life.^56^ Such assumptions could inadvertently lower HSUVs for CAD, contrasting with the nuanced realities of these individuals' lives.^51^ This systemic bias necessitates a re-evaluation of how HSUVs are conceptualized and measured, ensuring they capture the lived experiences of those with disabilities, rather than relying on normative expectations of function and health.

Despite limitations, noteworthy findings emerged regarding the variability of HSUVs across different disability types. The lowest reported HSUVs, reaching as low as −0.13, were observed in children with primary physical disabilities.^25^ Conversely, children with sensory impairments, autism spectrum disorders, intellectual impairments, and speech or language disorders generally exhibited HSUVs greater than 0.7.^20^, ^21^, ^22^, ^23^, ^24^^,^^26^^,^^35^, ^36^, ^37^, ^38^^,^^41^^,^^42^^,^^46^ Interestingly, children with primary physical impairments consistently demonstrated lower HSUVs compared with those with other impairments, with mean values ranging from −0.13 to 0.95.^25^^,^^34^ Although there were some similarities in reported HSUVs within each disability group, considerable variation was observed across different measures and severity levels. These findings underscore the nuanced impact of disability on a child's HRQoL, influenced by factors such as the nature and severity of the disability, as well as the specific measures used.

Our quality assessment revealed a need to improve the methodological quality of studies assessing HSUVs, aligning with conclusions of other systematic reviews.^58^, ^59^, ^60^ However, our assessment is limited by the absence of systematic evaluation tools to assess the methodologic quality of utility studies. A need for comprehensive tools has been recognized.^61^ Other limitations of this review include the exclusion of non–peer-reviewed publications such as unpublished theses and conference presentations, and non-English studies, which may have included relevant information. The limited number of studies for each disability type and the heterogeneity in the studies, such as differences in the instruments used, also contribute to challenges in interpreting the results, making it difficult to generalize the findings to other populations with similar disabilities.

Health utility assessments have rarely been conducted or published in low and middle-income settings, making it unclear whether preference values from high-income settings would apply in those countries. In addition, future research is required to evaluate the impact of the methods used for deriving preference values to enhance their effectiveness. In summarizing the instruments validated for use in this population group, we have also identified those that have not yet been validated. We hope that the identification of these knowledge gaps will encourage and direct future instruments validation research efforts.

## Conclusions

This systematic review provides valuable insights into the measurement of HSUVs in children and adolescents with diverse disability types, holding significant implications for health policy and decision-making, particularly in economic evaluations of health care interventions for CAD. Careful selection of appropriate methodologies and respondents to elicit HSUVs for CAD is crucial to ensure the collected data is reliable and effectively informs decisions aimed at enhancing their lives. Priority should be placed on the developing and validating HSUV measures tailored to CAD, which are devoid of ableist biases and truly reflective of unique health experiences. We further advocate for methodologies that capture a broad spectrum of health states specific to diverse disabilities, fostering the development of measures that advance, rather than hinder, health equity.

## CRediT authorship contribution statement

**Lucy Kanya:** Writing – review & editing, Writing – original draft, Validation, Supervision, Project administration, Methodology, Investigation, Funding acquisition, Formal analysis, Data curation, Conceptualization. **Nana Anokye:** Writing – review & editing, Writing – original draft, Validation, Methodology, Funding acquisition, Formal analysis, Conceptualization. **Ahmad Hecham Alani:** Writing – review & editing, Writing – original draft, Visualization, Validation, Project administration, Methodology, Investigation, Formal analysis. **Nandini Jayakumar:** Writing – review & editing, Validation, Formal analysis, Data curation. **Jennifer M. Ryan:** Writing – review & editing, Writing – original draft, Validation, Supervision, Methodology, Formal analysis, Conceptualization.

## Declaration of Competing Interest

This review was supported by a Research Catalyst Award from the Institute of Environment, Health and Societies, 10.13039/100008475Brunel University London and a Researcher Development Fund from the London School of Economics and Political Science. None of the funders was involved in the design and conduct of the study. The authors have no conflicts of interest to declare.

## Figures and Tables

**Appendix:** Quality assessment checklist

Reference	Year	1. Was the study aim and objectives clearly specified?	2. Was the sample comprehensively described	3. Was the sample representative of the target population as described in the aim	4. Were the inclusion and exclusion criteria explicit?	5. Was the condition described including severity?	6. Was the recruitment of study participants well defined?	7. Are response rates reported?	8. Was the sample size justified?	9. Was sufficient detail provided regarding the administration of the method	10. Were assessors trained in the administration of the method if appropriate	11. Were reasons for missing data explained?	12. Do the authors discuss potential sources of bias and attempt to minimize bias (eg, by adjusting for confounding)?	13. Do the authors discuss if the findings are generalizable to the source population (ie, externally valid?)	14. Do authors declare potential conflicts of interest?	Overall score
Cheng et al^18^	2000	Yes	Yes	Yes	Yes	Yes	Yes	Yes	Not clear	Yes	Not clear	No	Yes	SW∗	Yes	10.5
Tilford et al^19^	2005	Yes	Yes	Yes	Yes	Yes	SW	Yes	SW	Yes	Not clear	SW	Yes	Yes	Yes	11.5
Barton et al^20^	2006	Yes	SW	Yes	Yes	Yes	Not clear	Yes	Not clear	Yes	Not clear	Yes	Not clear	SW	Yes	9
Sach et al^21^	2007	Yes	SW	Yes	SW	No	Not clear	Not clear	Yes	SW	No	No	Yes	No	Yes	6.5
Rosenbaum et al^45^	2007	Yes	SW	Yes	Yes	No	SW	Yes	Not clear	Yes	Yes	Yes	No	No	Yes	9
Smith-Olinde et al^22^	2008	Yes	Yes	Yes	Yes	Yes	Yes	Yes	Not clear	Yes	Not clear	Yes	Yes	Yes	Yes	12
Petrou et al^24^	2009	Yes	Yes	Not clear	Yes	Not clear	Not clear	Yes	Not clear	SW	Not clear	Yes	Yes	Yes	Yes	8.5
Carroll et al^23^	2009	Yes	Yes	Yes	SW	Yes	Yes	Yes	Yes	Yes	Yes	No	SW	No	Yes	11
Young et al^25^	2010	Yes	SW	Yes	No	Yes	SW	Yes	Yes	No	No	No	No	No	Yes	7
Tilford et al^26^	2012	Yes	Yes	Yes	Yes	Yes	SW	Yes	Not clear	Yes	Not clear	SW	Not clear	Yes	Yes	10
Petrou et al^27^	2013	Yes	Yes	Yes	Yes	Yes	Not clear	Yes	Not clear	Yes	Not clear	No	Yes	No	No	8
Kulpeng et al^28^	2013	Yes	Yes	Not clear	Yes	Yes	Yes	Yes	Not clear	Yes	Yes	No	Yes	SW	Yes	10.5
Domellöf et al^30^	2014	Yes	SW	Yes	Yes	No	SW	Yes	Not clear	Yes	Not clear	SW	No	Yes	Yes	8.5
Burström et al^29^	2014	Yes	Yes	No	SW	Yes	Yes	Yes	SW	Yes	No	Yes	No	Yes	No	9
Chevreul et al^31^	2015	Yes	SW	Yes	Yes	No	Yes	Yes	Not clear	Yes	Not clear	No	No	Yes	Yes	8.5
Landfeldt et al^33^	2016	Yes	SW	No	Yes	Yes	Yes	Yes	SW	Yes	Not clear	Not clear	No	Yes	yes	9
Chevreul et al^32^	2016	Yes	SW	Yes	Yes	No	Yes	Yes	Not clear	Yes	Not clear	No	Yes	Yes	Yes	9.5
Hind et al^34^	2017	Yes	SW	Yes	Yes	No	Yes	Not clear	Yes	SW	Not clear	Not clear	SW	Yes	Yes	8.5
Ramanan et al^35^	2019	Yes	Yes	SW	Yes	Yes	Yes	SW	Yes	Yes	SW	Not clear	No	Yes	Yes	10.5
Le et al^36^	2019	Yes	Yes	Yes	Yes	Yes	Yes	Not clear	SW	Yes	Not clear	Not clear	Yes	SW	No	9
Kirkham et al^37^	2020	Yes	SW	Yes	Yes	Yes	Yes	Yes	Yes	Yes	Not clear	SW	SW	Not clear	Yes	10.5
Tonmukayakul et al^39^	2020	Yes	Yes	SW	Not clear	Yes	Not clear	No	Not clear	Yes	Not clear	Yes	Not clear	SW	No	6
Nair et al^38^	2020	Yes	SW	SW	Yes	Yes	Not clear	No	Not clear	Not clear	Not clear	No	No	No	No	4
Liu et al^40^	2021	Yes	SW	SW	Not clear	Yes	SW	No	No	Yes	Not clear	Not clear	No	Yes	No	5.5
Randell et al^41^	2022	Yes	Yes	Yes	Yes	Yes	Yes	Not clear	Yes	Yes	SW	Yes	Yes	Yes	No	11.5
Khan et al^43^	2023	Yes	Yes	Yes	Yes	Yes	Not clear	Not clear	Yes	SW	Yes	Not clear	SW	SW	Yes	9.5
van Westrhenen et al^42^	2023	Yes	Yes	Yes	Yes	Yes	Not clear	Yes	Yes	SW	Yes	Yes	SW	No	Yes	11
Da Costa et al^44^	2023	Yes	Yes	Yes	Yes	SW	Yes	Not clear	Yes	Yes	Yes	Not clear	No	No	No	8.5
Downs et al^47^	2024	Yes	SW	Yes	Yes	Yes	Yes	Yes	No	Yes	Yes	Not clear	No	Yes	Yes	10.5
Blackmore et al^46^	2024	Yes	SW	Yes	Yes	Yes	Yes	Yes	No	Yes	Yes	Not clear	No	Yes	Yes	10
Bukhari and Zawawi^48^	2024	Yes	SW	Yes	Yes	Yes	Yes	Yes	No	Yes	SW	Not clear	SW	Yes	Yes	10

*SW*, Somewhat.
